# UBA2 activates Wnt/β-catenin signaling pathway during protection of R28 retinal precursor cells from hypoxia by extracellular vesicles derived from placental mesenchymal stem cells

**DOI:** 10.1186/s13287-020-01943-w

**Published:** 2020-10-02

**Authors:** Kyungmin Koh, Mira Park, Eun Soo Bae, Van-An Duong, Jong-Moon Park, Hookeun Lee, Helen Lew

**Affiliations:** 1 Department of Ophthalmology, CHA Bundang Medical Center, CHA University, Seongnam, Republic of Korea; 2 Department of Ophthalmology, Kim’s Eye Hospital, Konyang University College of Medicine, Seoul, Republic of Korea; 3 Gachon Institute of Pharmaceutical Sciences, Gachon College of Pharmacy, Gachon University, Incheon, Republic of Korea

**Keywords:** Exosome, hPMSCs, Optic nerve injury, Retinal precursor cells, UBA2, Wnt/β-catenin

## Abstract

**Background:**

Stem cell transplantation has been proposed as an alternative treatment for intractable optic nerve disorders characterized by irrecoverable loss of cells. Mesenchymal stem cells, with varying tissue regeneration and recovery capabilities, are being considered for potential cell therapies. To overcome the limitations of cell therapy, we isolated exosomes from human placenta-derived mesenchymal stem cells (hPMSCs) and investigated their therapeutic effects in R28 cells (retinal precursor cells) exposed to CoCl_2_.

**Method:**

After 9 h of exposure to CoCl_2_, the hypoxic damaged R28 cells were divided into the non-treatment group (CoCl_2_ + R28 cells) and treatment group (CoCl_2_ + R28 cells treated with exosome). Immunoblot analysis was performed for Pcna, Hif-1α, Vegf, Vimentin, Thy-1, Gap43, Ermn, Neuroflament, Wnt3a, β-catenin, phospo-GSK3β, Lef-1, UBA2, Skp1, βTrcp, and ubiquitin. The proteomes of each group were analyzed by liquid chromatography/tandem mass (LC-MS/MS) spectrometry. Differentially expressed proteins (DEPs) were detected by label-free quantification, and the interactions of the proteins were examined through signal transduction pathway and gene ontology analysis.

**Result:**

We observed that exosome could significantly recover proliferation damaged by CoCl_2_ treatment. In addition, the treatment group presented the decreased expression of Hif-1α protein (*P* < 0.05) and increased expression of proliferation marker, Pcna, and nerve regeneration-related factors such as Vimentin, Thy-1, and Neuroflament (*P* < 0.05) compared with the non-treatment group. In total, 200 DEPs were identified in the non-treatment group and treatment group (fold change ≥ 2, *p* < 0.05). Catenin and ubiquitin systems (UBA2, UBE2E3, UBE2I) were found in both the DEP lists of downregulated proteins from the non-treatment group and upregulated proteins from the treatment group. The mRNA expressions of ubiquitin systems were significantly decreased under hypoxic conditions. Moreover, UBA2 and Wnt/β-catenin protein were associated with the rescue of the hypoxic damaged R28 cells. Using a siRNA system, we could find it out that hPMSC exosomes could not repair altered expressions of target proteins by CoCl_2_ in lacking UBA2 R28 cells.

**Conclusion:**

This study reported that hypoxic damaged expression of regeneration markers in R28 cells was significantly recovered by hPMSC exosomes. We could also demonstrate that UBA2 played a key role in activating the Wnt/β-catenin signaling pathway during protection of hypoxic damaged R28 cells, induced by hPMSC exosomes.

## Background

Optic neuropathies are the most common cause of irreversible vision loss [1]. There is still no effective treatment for optic neuropathies; the improvement of new treatment methods is required. In previous studies, neural regeneration has been induced by injecting placenta-derived mesenchymal stem cells (MSCs) [2]. MSCs are multipotent stromal cells that exist in mesenchymal tissues; their neuroprotective effect in optic nerve (ON) injury models has been reported [3].

MSCs have some exclusive features compared to other stem cell types. They are simple to isolate and expand and exhibit a high differentiation capacity, low immunological response, and low risk of tumor formation [4, 5]. However, direct injection of MSCs is highly prone to several issues, including the risk of thrombosis and tumor-related mutations [6]. Concerns have been raised about the safety of MSCs for clinical use, with studies reporting the possible threat of in vitro MSCs to develop tumors, ectopic tissue formation, toxicity caused by cells, and immune-related rejection on transplantation [7, 8]. Mice injected with these MSCs developed tumors in multiple organs, since chromosome instability and elevated telomerase activity were proposed as contributing factors for developing malignancy in mouse MSCs [9]. Multifocal organizing thrombi were noted in the pulmonary arteries 1 week after the administration of a single intravenous injection of mesenchymal stem cells [10]. On the other hand, MSC-secreted exosomes are smaller and easier to produce, and they appear no risk of tumor formation [11]. Therefore, to lower this risk, we paid attention to the nanosized extracellular vesicles (EV), exosomes, which are smaller.

Exosomes are small lipid-bound cellularly secreted vesicles that comprise ectosomes secreted directly from plasma membranes and apoptotic bodies released from dying cells [12]. They are a type of membrane vesicle, with a size less than 150 nm, containing proteins, mRNA, and miRNA, and are derived from internal vesicles of multivesicular bodies such as tumor cells, T cells, and mast cells [13]. Exosomes mediate some of the tissue-healing properties of MSCs, are helpful in tissue regeneration, and present strong therapeutic potential [14] for diseases such as myocardial infarction, spinal cord injury, Alzheimer’s disease, and diabetes [15, 16]. They are not self-replicating and, owing to their small size, can be sterilized by filtration, which makes them promising for therapeutics [17, 18].

Previous studies of the effect of MSC and MSC-derived EVs provided conflicting results [19–21]. A report demonstrated that exosomes from MSCs have a similar role in promoting tumor growth to the MSCs themselves [19]. MSC-derived exosomes exhibited different protein and RNA profiles compared with their donor cells, and these vesicles could be internalized by breast cancer cells. The results demonstrated that MSC-derived exosomes significantly downregulated the expression of vascular endothelial growth factor (VEGF) in tumor cells, which lead to inhibition of angiogenesis [20]. However, no studies comparing the effects of MSC and MSC exosome in the ON precursor cell damage were reported. The factors and mechanism to rescue damaged ON precursor cells would be different in each type of exosome. Therefore, we isolated exosomes from placenta-derived MSCs and conducted a study to verify their neuro-regeneration effect.

## Methods

### Human placenta-derived mesenchymal stem cell (hPMSC) preparation and isolation of exosomes from hPMSCs

Human placenta stem cells were obtained from CHA General Hospital, Seoul, Republic of Korea. The sample collection and use for research purposes were approved by the Institutional Review Board of the hospital. Preparation and culturing were conducted as previously reported [22]. Human PMSCs were cultured in Minimum Essential Medium (MEM)-alpha GlutaMAX (Thermo Fisher Scientific, Waltham, MA, USA) supplemented with 10% FBS (Thermo Fisher Scientific), 1% penicillin/streptomycin (Thermo Fisher Scientific), 25 ng/mL human fibroblast growth factor 4 (Peprotech Inc., Rocky Hill, NJ, USA), and 1 μg/mL heparin (Sigma-Aldrich, St. Louis, MO, USA). When 80% confluence was reached, the culture medium was replaced with MEM-alpha GlutaMAX containing 10% exosome-free FBS (Thermo Fisher Scientific). The conditioned hPMSCs were harvested from the medium, and residual cells and debris were discarded by centrifuging at 2000×*g* for 10 min at 4 °C. After centrifugation, the supernatant was filtered using a 0.2-μm pore filter and transferred to a centrifuge tube (Pall Corporation, Port Washington, NY, USA). The supernatant was centrifuged at 4000 rpm for 45 min at 4 °C. The collected supernatant again underwent ultracentrifugation at 27,500 rpm for 85 min at 4 °C. Thereafter, the supernatant was removed, and the precipitate was washed with phosphate-buffered saline (PBS) and then ultracentrifuged at 27,500 rpm for 85 min at 4 °C. Finally, the exosome precipitates were dissolved in 100 μL PBS and quantified by the BCA method. The exosomes were stored at − 80 °C.

### Cell culture and treatment

R28 retinal precursor cells were provided from Dr. Seigel [23]. Immortalized R28 retinal precursor cells were maintained in low-glucose Dulbecco’s modified Eagle’s medium (DMEM; Sigma-Aldrich) with 10% FBS (Thermo Fisher Scientific), 1× minimal essential medium nonessential amino acids (Thermo Fisher Scientific), 100 μg/mL gentamicin (Sigma-Aldrich), and 1% penicillin/streptomycin (Thermo Fisher Scientific). A hypoxic condition was induced by exposing the cells to CoCl_2_ (Sigma-Aldrich). R28 cells (2 × 10^5^) were treated with CoCl_2_ (200 μM) for 9 h. Then, they were treated with hPMSC exosomes (12 μg/mL). After 24 h, the cells were harvested and prepared for analysis.

### Small interfering RNA

The target sequence of siRNA (Bioneer Corporation, Daejeon, Republic of Korea) was as follows: siRNA rat UBA2, 5′- GCA CGA AAC CAU GUG AAU AGG A. siRNA negative control (Bioneer) was used as the negative control (scramble). R28 cells were transfected using Lipofectamine 3000 (Thermo Fisher Scientific) according to the manufacturer’s instructions.

### Cell Counting Kit-8 (CCK-8) assay

R28 cells were plated in 96-well plates at the density of 1 × 10^4^ cells/well. After CoCl_2_ (200 μM) treatment for 9 h, the cells were incubated with hPMSC exosomes (12 μg/mL). After 24 h, R28 cells were incubated with 10 μL CCK-8 (Dojindo Laboratories, Munich, Germany) for 1 h. The absorbance at 450 nm was detected by a microplate reader (Molecular Devices, San Jose, CA, USA).

### Reverse transcription-polymerase chain reaction (RT-PCR) analysis

Total RNA was isolated from hOFs using TRIzol reagent (Thermo Fisher Scientific). RT-PCR was performed with nPfu-Forte PCR polymerase (Enzynomics, Daejeon, Republic of Korea). We quantified the gene expression using ImageJ software (National Institutes of Health, Bethesda, MD, USA), and RT-PCR reactions were performed using a CFX-96 machine (Bio-Rad Laboratories, Hercules, CA, USA). The nucleotide sequences of all the primers used were as follows: Rat *UBA2* FP: 5′- ACG ATT CGG AAC ACA CCT TC, RP: 5′- GCT TCA GCC TCT GTT GGT TC; Rat *UBE2E3* FP: 5′- TCG AGT GCT GTG TTC AAA GG, RP: 5′- CTG GTG CTA GGG CTC TCA TC; Rat *UBE2I* FP: 5′- TCT CCC TGC CTG TTA GCT GT, RP: 5′- TGG GCT GTA GGG TAA GGT TG.

### Immunoblot analysis

R28 cells were lysed in radioimmunoprecipitation assay (RIPA) buffer. Equal amounts of total protein were resolved by sodium dodecyl sulfate-polyacrylamide gel electrophoresis (SDS-PAGE) and transferred to membranes. The membranes were immunoblotted with anti-Pcna (Agilent Technologies, Santa Clara, CA, USA), Hif-1α (Abcam, Cambridge, UK), Vegf, Thy-1, Gap43 (Santa Cruz Biotechnology, Santa Cruz, CA, USA), Ermn (Abcam), Neuroflament (Cell Signaling Technology, Danvers, MA, USA), Vimentin, Wnt3a, β-catenin (GeneTex, Irvine, CA, USA), phospo-GSK3β (Cell Signaling Technology), Lef-1 (GeneTex), UBA2 (Abcam), Skp1, βTrcp (Cell Signaling Technology), and ubiquitin (Abcam). After washing, the membranes were incubated at room temperature for 2 h with horseradish peroxidase-conjugated anti-rabbit/mouse/goat IgG secondary antibodies at a 1:10,000 dilution (GeneTex). Immunoreactive bands were visualized with enhanced chemiluminescence solution (Bio-Rad Laboratories) and analyzed using ImageQuant™ LAS 4000 (GE Healthcare, Chicago, IL, USA).

### Proteomics

Proteomic analyses were performed for 4 types of samples: R28 cells with PBS, R28 cells with exosomes, R28 cells treated with CoCl_2_, and R28 cells treated with CoCl_2_ and exosomes. Thus, we investigated how exosomes worked in both undamaged and damaged cells.

#### Materials

Tris (2-carboxyethyl) phosphine (TCEP) was supplied by Thermo Fisher Scientific. Formic acid (FA) and iodoacetamide (IAA) were purchased from Sigma-Aldrich. Trypsin was obtained from Promega (Madison, WI, USA). High-performance liquid chromatography (HPLC)-grade water and acetonitrile were purchased from JT Baker (Phillipsburg, NJ, USA).

#### Sample preparation

R28 cells (2 × 10^5^) were treated with CoCl_2_ (200 μM) for 9 h. Then, they were treated with hPMSC exosomes (12 μg/mL). After 24 h, the cells were harvested. Each cell pellet then was mixed with 1 mL of lysis solution (8 M Urea, 0.1 M Tris-HCl buffer, pH 8.5) and 40 μL of protease inhibitor cocktail (× 25 stock solution) in glass tubes. Cell lysis was performed using a Covaris S2 Focused-Ultrasonicator (Covaris, Woburn, MA, USA) for 8 min. The protein concentrations in the samples were determined using Pierce BCA Protein Assay Kits (Thermo Fisher Scientific). Filter-aided sample preparation (FASP) was performed using Ultracel® YM-30 centrifugal filters (Merck Millipore, Germany), as previously reported [24]. In brief, protein (100 μg) was reduced with TCEP (37 °C, 30 min), alkylated with IAA (25 °C, 30 min, in the dark), and digested with trypsin (37 °C, 18 h, enzyme to protein ratio = 1:50). After digestion, the peptide mixtures were collected. FA was added to inactivate trypsin. The samples were then desalted using C18 Micro spin columns (Harvard Apparatus, MA, USA), vacuum-dried (1800 rpm, 3 h, ScanSpeed 40 centrifugal evaporator), and reconstituted in 0.1% FA/water (solvent A) prior to analysis.

#### Liquid chromatography-tandem mass spectrometry (LC-MS/MS) analysis

An LC-MS/MS setup consisting of a Dionex Ultimate 3000 HPLC system coupled with a Q Exactive™ Hybrid Quadrupole-Orbitrap MS (Thermo Fisher Scientific) system was used for sample analysis. Samples were loaded into an Acclaim™ PepMap™ 100 C18 nano-trap column (75 μm × 2 cm, 3 μm particles, 100 Å pores, Thermo Fisher Scientific) using solvent A at a flow rate of 2.5 μL/min for 5 min. An Acclaim™ PepMap™ C18 100A RSLC nano-column (75 μm × 50 cm, 2 μm particles, 100 Å pores, Thermo Fisher Scientific) was used to separate the peptide mixtures. The solvent consisted of solvent A and solvent B (0.1% FA/80% ACN). The flow rate was fixed at 300 nL/min. A 185-min gradient setup for solvent B was used as follows: 4% (14 min), 4–20% (61 min), 20–50% (81 min), 50–96% (1 min), 96% (10 min), 96–4% B (1 min), and 4% (17 min). The nano-electrospray ionization source was operated in positive mode with a spray voltage of 2.0 kV. The capillary temperature was 320 °C. The isolation width was ± 2 m/z, and the scan range was 400–2000 m/z. The resolutions in full-MS scans and MS/MS scans at 200 m/z were 70,000 and 17,500, respectively. MS was conducted using a data-dependent acquisition method. The top ten precursor ions with the highest intensity were isolated in the quadrupole and fragmented by higher-energy collisional dissociation with 27% normalized collisional energy. Dynamic exclusion was set at 20 s to minimize repeated analyses of the same abundant precursor ions.

#### Data processing and bioinformatics

Database search for proteins and data processing were conducted as previously reported [25]. In brief, raw MS/MS data files were searched against a SwissProt human protein database (https://www.uniprot.org/) using a built-in Andromeda search engine in MaxQuant version 1.5.8.3 (www.coxdocs.org) for label-free quantification (LFQ). The following parameters were used for the search: missed cleavages with trypsin, ≤ 2; variable modifications, methionine oxidation (+ 15.995 Da), and carbamylation of protein in N-term (+ 43.0006 Da); static carbamidomethylation of cysteine (+ 57.0215 Da); first search peptide tolerance, 20 ppm; and main search peptide tolerance, 4.5 ppm. A false discovery rate (FDR) cutoff of 1% was used. LFQ data from MaxQuant were imported into Perseus software platform version 1.6.5.0 (www.coxdocs.org). Protein LFQ intensities were transformed using log2(*x*), and samples with missing values for given proteins were assigned random values using the imputation principle (downshift 1.8, width 0.3, total matrix mode). After *Z*-score normalization, Student’s *T* test was used to compare the protein abundances of the groups. Differentially expressed proteins (DEPs) were filtered with a cutoff *p* value ≤ 0.05 and log2FC ≥ 1 (fold-change). Heatmap was generated. Gene ontology (GO) analysis was performed using Panther (http://geneontology.org/). Kyoto Encyclopedia of Genes and Genomes (KEGG) pathways and protein-protein interactions were analyzed using the String database (https://string-db.org/).

### Statistical analyses

All the results are presented as mean ± standard error of the mean (SEM). Data analyses were conducted using GraphPad Prism (GraphPad, La Jolla, CA, USA). Statistically significant differences were identified using the *t* test or nonparametric statistical test, followed by the Mann-Whitney *U* test at a significance level of 5%.

## Results

### Characterization and recovery effects of hPMSC exosomes

**Fig. 1:**
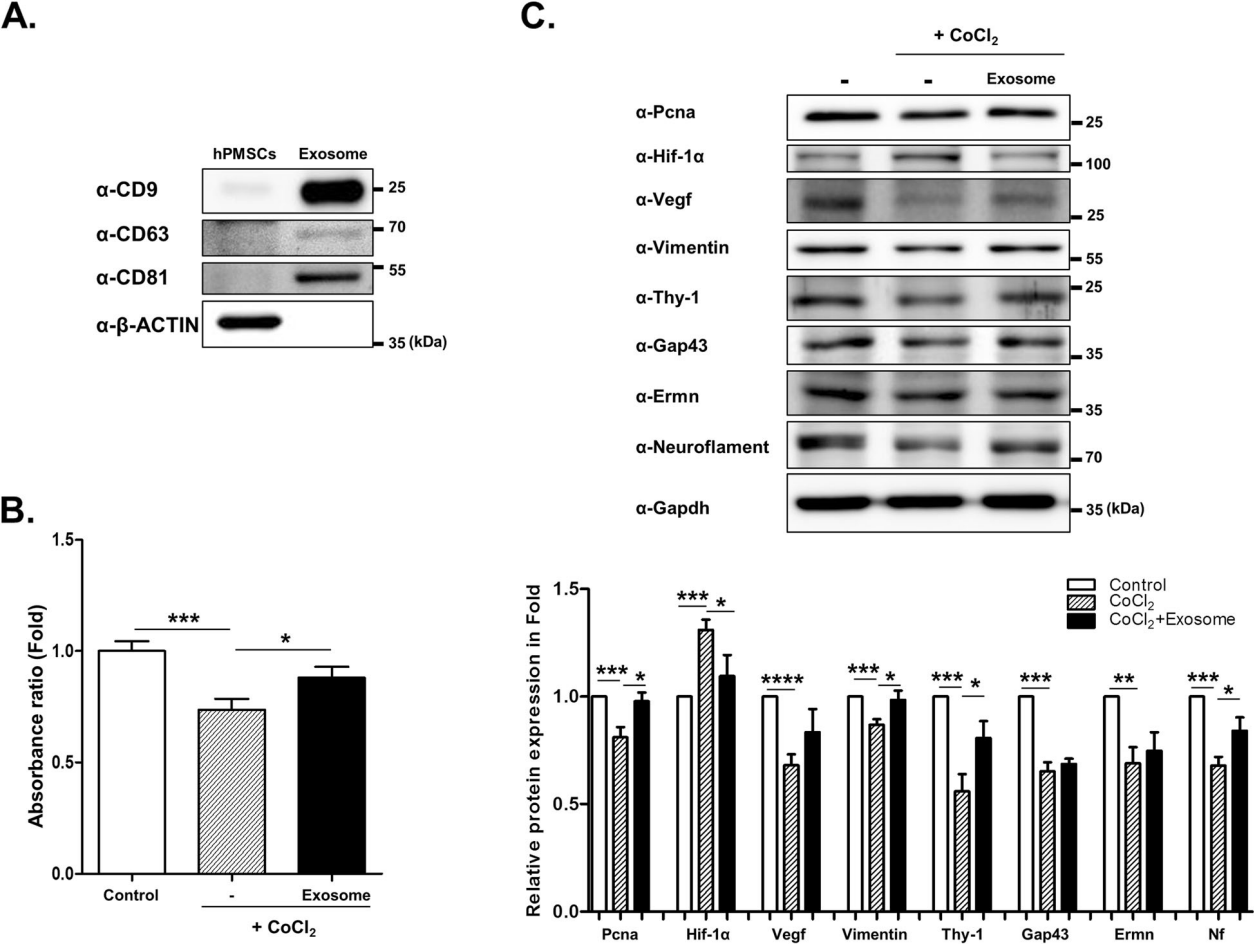
Characterization and recovery effects of hPMSC-derived exosomes. **a** Isolated exosome protein expressing exosomal markers CD9, CD63, and CD81. R28 cells were treated with CoCl_2_ (200 μM). After incubation for 9 h, the cells were treated with exosomes. **b** CCK-8 assays performed after 24 h. Data are presented as mean ± SEM (***p < 0.0001; *p < 0.05). **c** Western blot analyses of target protein expression levels, using R28 lysates with CoCl_2_. The quantified values of target protein expression are presented (bottom panel). Statistical significance was determined using an unpaired *t* test (*p < 0.05; **p < 0.005; ***p < 0.0005; ****p < 0.0001). All experiments were performed in triplicate

The isolated exosome protein expressed the exosomal markers CD9, CD63, and CD81 (Fig. 1a). To examine the changes in the target proteins of R28 cells under hypoxic conditions, we exposed the cells to CoCl_2_. After 9 h, the hypoxia-damaged R28 cells were treated with hPMSC exosomes. We performed CCK-8 assay to determine the effects of the exosomes on CoCl_2_-induced cell proliferation. The cell proliferations were significantly recovered upon the administration of the exosomes (Fig. 1b). The exosomes also restored the target protein expression disturbed by CoCl_2_. Hif-1α expression, which increased after CoCl_2_ exposure, was significantly decreased by the exosome treatment (Fig. 1c). To support the proliferation assay result, we investigated the changes of proliferation-related gene expression. The CoCl_2_-induced downregultaion of proliferating cell nuclear antigen (PCNA) was significantly increased by exosome treatment (Fig. 1c). In contrast to that of Hif-1α, the expression of regeneration-related proteins such as Vegf, Vimentin, Thy-1, Gap43, Ermn, and Neuroflament decreased under hypoxic conditions. The expression of Vimentin, Thy-1, and Neuroflament significantly increased after the exosome treatment (Fig. 1c).

### Hierarchical clustering and gene ontology

**Fig. 2:**
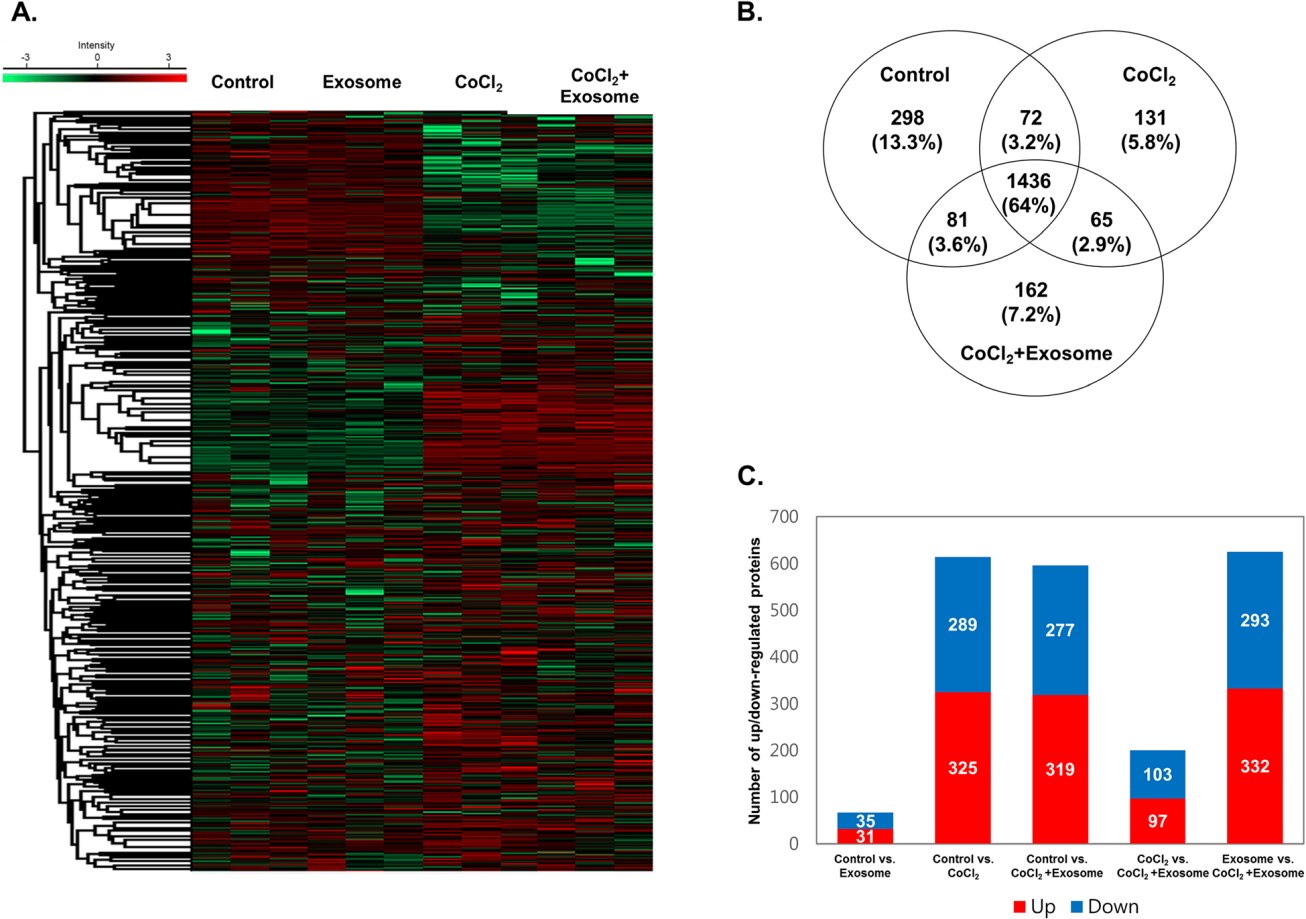
Cluster analysis of differentially expressed proteins in R28 cells. MaxQuant version 1.5.8.3 (www.coxdocs.org) for label-free quantification (LFQ) was used to identify differentially expressed proteins in treated groups (exosome, CoCl_2_, and CoCl_2_ + exosome) and PBS-treated control cells. **a** Heatmap of differentially expressed proteins (*p* ≤ 0.05 and log2FC ≥ 1) in lysates of four groups (control, exosome, CoCl_2_, and CoCl_2_ + exosome) analyzed by hierarchical clustering. High expression is shown in red; low expression is shown in green. **b** Venn diagram showing the number of proteins identified in proteomic analysis of experimental groups of R28 cells. **c** Graph presenting the number of significantly up- and downregulated proteins in experimental groups (*p* < 0.05)

To understand the integrated biological effects of exosome treatment during hypoxia, we performed proteomic analysis using R28 cells. The hierarchical clustering of the differentially expressed proteins was determined using four conditions (Control, Exosome, CoCl_2_, CoCl_2_ + exosome) (Fig. 2a). The Venn diagram in Fig. 2b shows the number of expressed proteins associated with injury and recovery processes. The control, CoCl_2_, and CoCl_2_ + exosome groups expressed 1887, 1704, and 1744 proteins, respectively, indicating an altered expression after exposure to CoCl_2_ (Fig. 2b). The number of differentially expressed proteins (DEPs) between each group indicated significant changes in the proteome under exposure to CoCl_2_ and CoCl_2_ + exosome (*p* < 0.05) (Fig. 2c).

### Gene ontology classification of reliably quantified proteins from exosomes in hypoxia-damaged retinal precursor cells

**Fig. 3:**
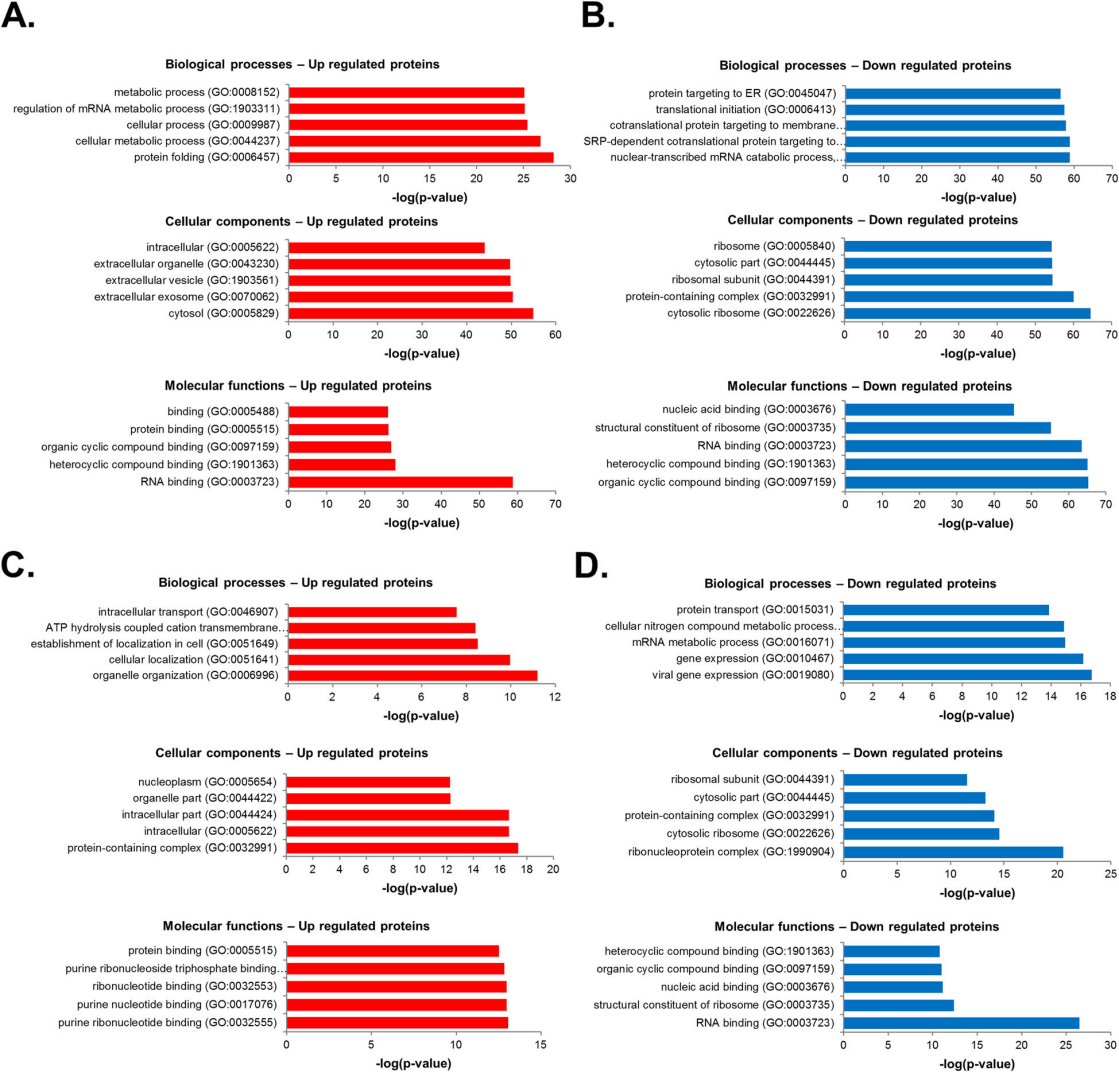
Gene ontology (GO) distribution of differentially expressed proteins in R28 cells damaged by CoCl_2_ and treated with hPMSC-derived exosomes. **a** GO annotations for biological processes, molecular functions, and cellular components of upregulated proteins in R28 cells damaged by CoCl_2_, compared with those of controls. **b** GO annotations for biological processes, molecular functions, and cellular components of downregulated proteins in R28 cells damaged by CoCl_2_, compared with those of controls. **c** GO annotations for biological processes, molecular functions, and cellular components of upregulated proteins in CoCl_2_ + R28 cells treated with exosomes, compared with those of R28 cells damaged by CoCl_2_. **d** GO annotations for biological processes, molecular functions, and cellular components of downregulated proteins in CoCl_2_ + R28 cells treated with exosomes, compared with those of R28 cells damaged by CoCl_2_ (false discovery rate ≤ 0.05)

In total, 614 DEPs were identified in R28 cells before and after exposure to CoCl_2_ (fold change ≥ 2, *P* < 0.05). Panther Classification System (version 15.0) was used for GO analysis of the DEPs. Using a false discovery rate ≤ 0.05, GO functional clusters were enriched and categorized into three databases: biological processes, molecular functions, and cellular components of the two groups. Figure 3 shows that the DEPs were classified as top 5 GO terms based on –log_10_ (*p* value). The upregulated proteins of CoCl_2_-treated R28 cells were involved in protein folding, cytosolic processes, and RNA binding, as shown in Fig. 3a. The downregulated proteins were related to nuclear-transcribed mRNA catabolic processes, cytosolic ribosomes, protein-containing complexes, RNA binding, and heterocyclic and organic cyclic compound binding (Fig. 3b). In total, 200 DEPs were identified in CoCl_2_ + R28 cells and CoCl_2_ + R28 cells treated with exosomes (fold change ≥ 2, *p* < 0.05). The upregulated proteins of CoCl_2_ + R28 cells treated with exosomes were involved in organelle organization, protein-containing complexes, intracellular components, purine ribonucleotides, and ribonucleotide binding, as shown in Fig. 3c. The downregulated proteins were related to gene expression, ribonucleoprotein complexes, and RNA binding (Fig. 3d).

### Network analysis of recovery process mediators

**Fig. 4:**
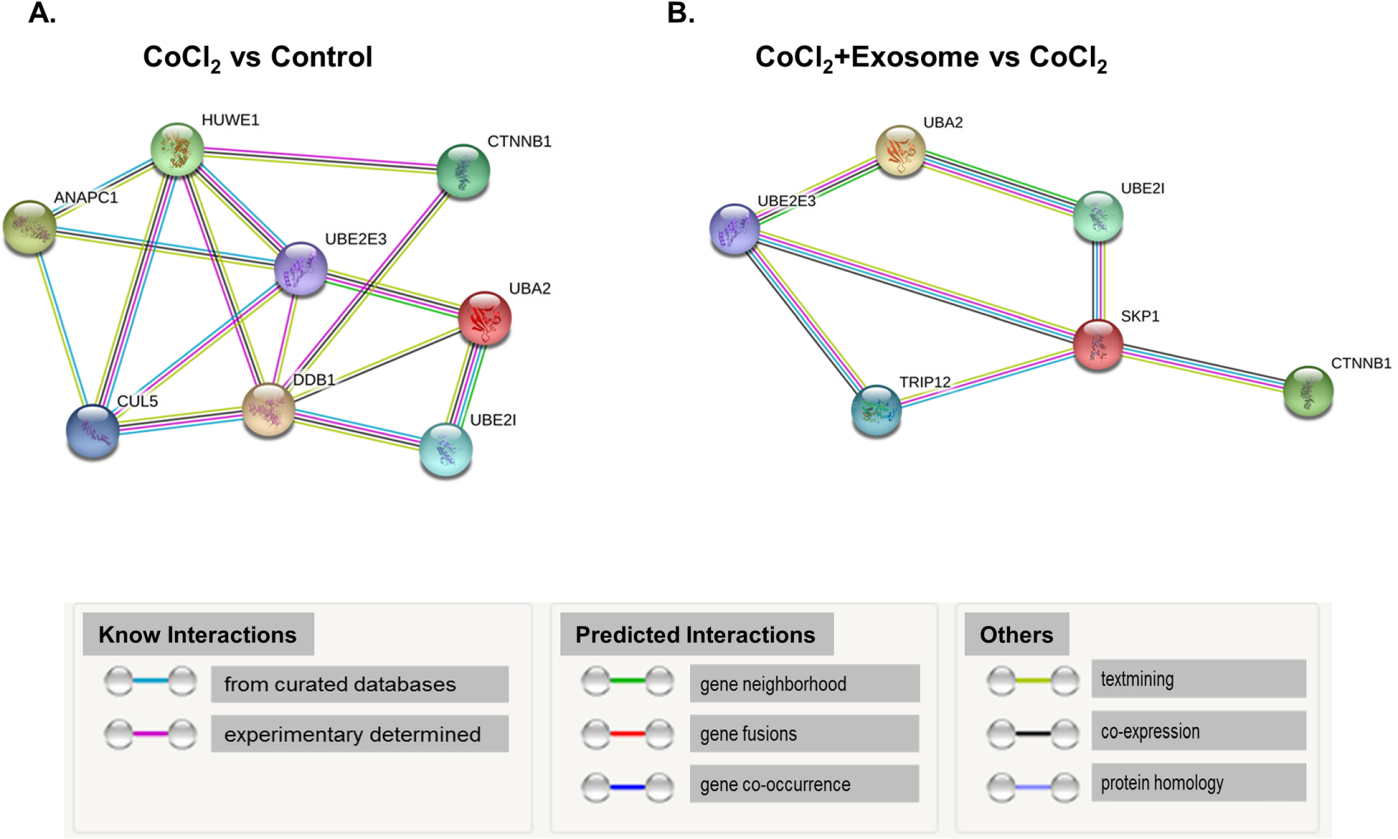
Network analysis of DEPs relating to ubiquitin-mediated proteolysis pathway in R28 cells. Protein-protein interactions in groups: **a** CoCl_2_ vs control and **b** CoCl_2_ + exosome vs CoCl_2_

We performed a detailed examination of the interactions of the proteins revealed by GO analysis. Proteins unique to both R28 cells damaged by CoCl_2_ and CoCl_2_ + R28 cells treated with exosomes were mainly involved in protein-containing complexes and RNA binding for molecular functions. Catenin and ubiquitin systems (UBA2, UBE2E3, UBE2I) were found in both the DEP lists of downregulated proteins from R28 cells damaged by CoCl_2_ and upregulated proteins from CoCl_2_ + R28 cells treated with exosomes. The interactions between them and other identified proteins of the ubiquitin-mediated proteolysis pathway are shown in Fig. 4a, b.

### Effects of hPMSC exosomes in hypoxia-damaged in vitro model

**Fig. 5:**
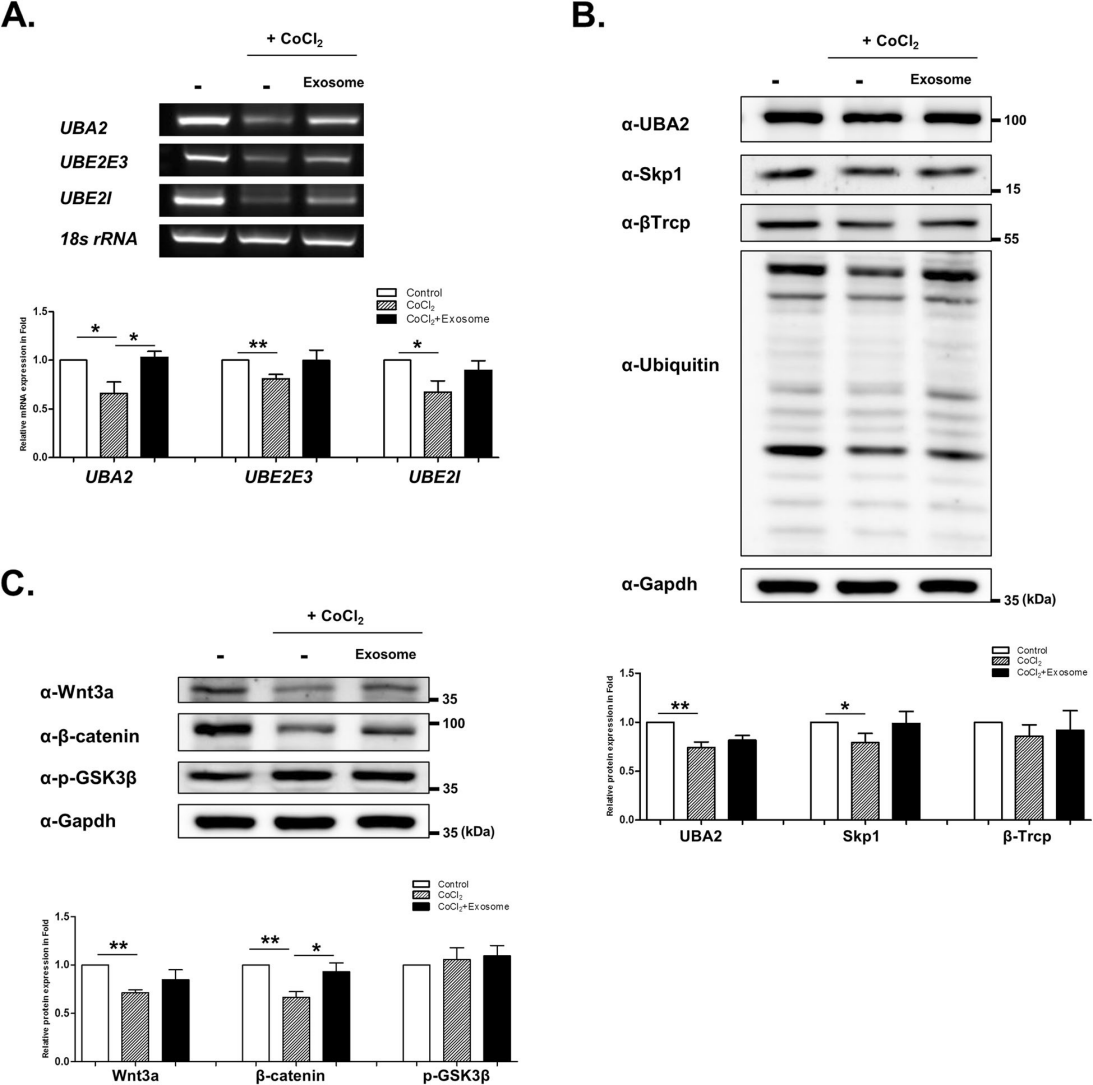
Effect of exosome treatment on target proteins altered by CoCl_2_. R28 cells were treated with CoCl_2_ (200 μM). After incubation for 9 h, the cells were treated with exosomes. **a** RT-PCR of target genes and Western blot analyses of **b** ubiquitin-proteasome system and **c** Wnt/β-catenin-related protein expression levels, performed using R28 lysates altered by CoCl_2_. Statistical significance was determined using an unpaired *t* test (*p < 0.05; **p < 0.005). All experiments were performed in triplicate

Based on proteomic data, we could verify significantly changed target proteins in both CoCl_2_ + R28 cells and CoCl_2_ + R28 cells treated with exosomes. To determine the relationship between CoCl_2_ and altered target protein expression, we determined the changes in exosome-induced expression during hypoxia, i.e., UBA2, UBE2E3, and UBE2I mRNA expression. These target genes from proteomic data were downregulated upon CoCl_2_ exposure. However, damaged UBA2 expression significantly recovered after exosome treatment (Fig. 5a). Furthermore, the expression of some proteins in the ubiquitin proteasome process, such as UBA2, Skp1, βTrcp, and ubiquitin, decreased in hypoxia-damaged R28 cells. The expression of the weakened proteins seemed to increase after exosome treatment, but it was not significant (Fig. 5b). CoCl_2_ treatment reduced Wnt3a and β-catenin protein expression. After exosome treatment, the expression increased. However, only the increase in β-catenin expression was significant (Fig. 5c).

### Role of UBA2 during recovery induced by hPMSC exosomes

**Fig. 6:**
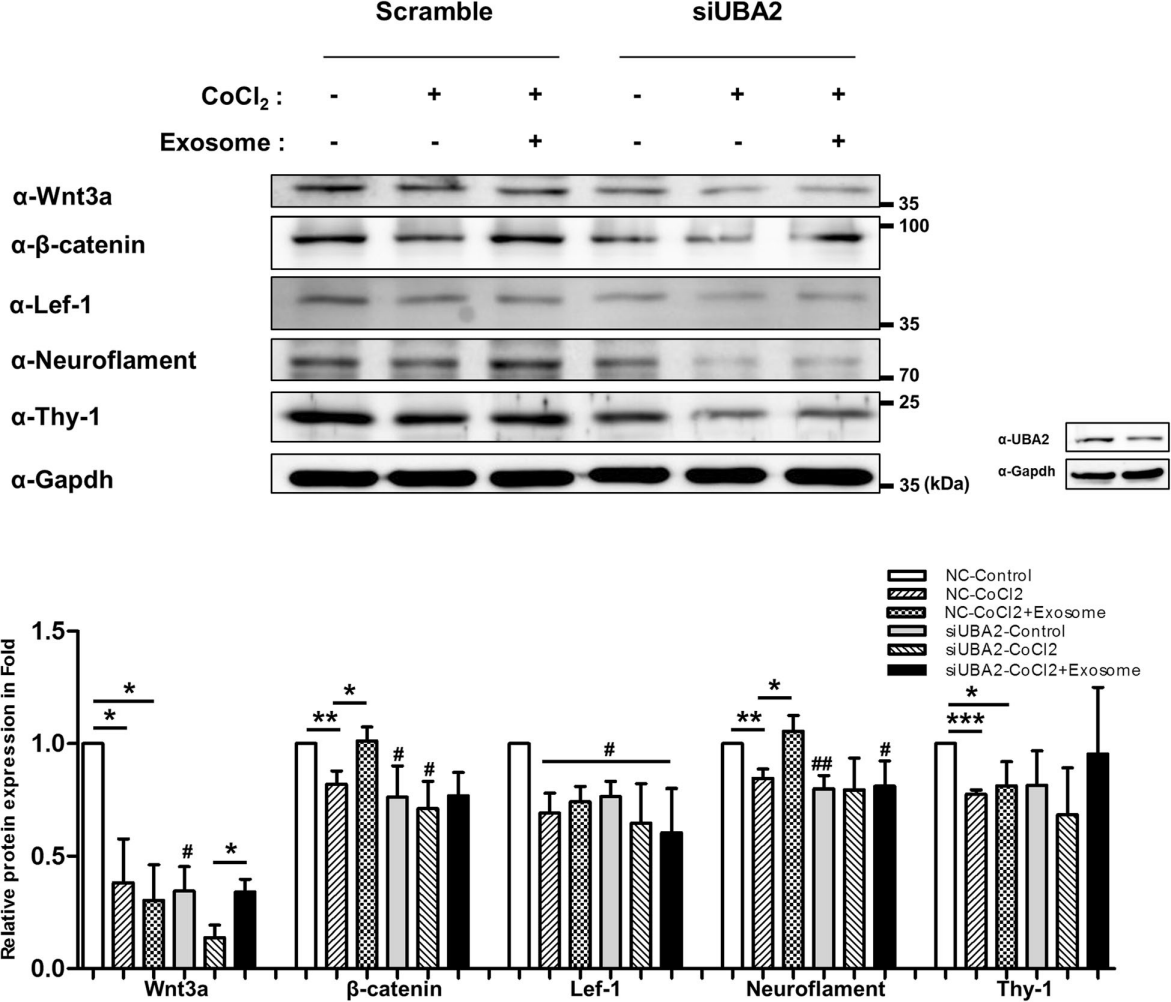
UBA2 mediation during recovery induced by exosomes. Scramble and siRNA (2 nM) targeting UBA2 were transfected into R28 cells. After incubation, UBA2 knockdown cells were exposed to CoCl_2_ (200 μM). After incubation for 9 h, the cells were treated with exosomes. Then, Western blot analyses were performed. The relative expression of target proteins was normalized with that in normal R28 cells. The results are expressed as mean ± standard error of mean (SEM). Statistical significance was determined using a nonparametric statistical test, followed by the Mann-Whitney *U* test (^#^*p* < 0.05, ^##^*p* < 0.005 vs. control with scramble; *p < 0.05, **p < 0.005, ***p < 0.0001). All experiments were performed in triplicate

**Fig. 7:**
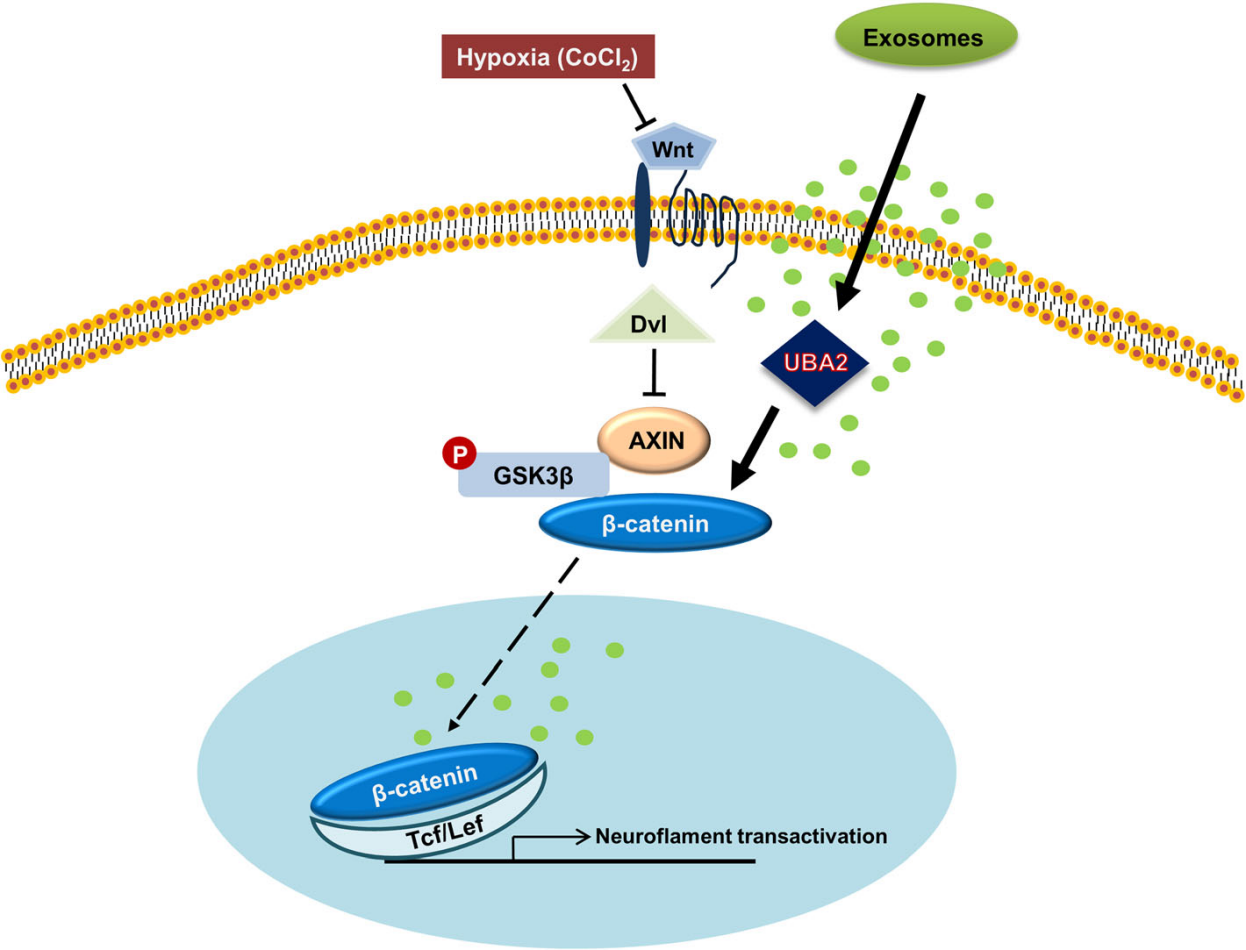
Proposed pathway of recovery of R28 cells, induced by hPMSC-derived exosomes, after hypoxic damage

Based on the proteomics and in vitro experiment results, we assumed that UBA2 was a mediator during the hypoxia recovery response induced by exosomes. Therefore, using an siRNA system, we investigated the effects of exosomes on UBA2-lacking R28 cells damaged by CoCl_2_. As shown in Fig. 6, in UBA2 knockdown R28 cells, the expression of β-catenin, Wnt3a, Neuroflament, and Thy-1 significantly reduced compared to that in scramble cells. Exosome treatment of cells damaged by CoCl_2_ restored the β-catenin, Neuroflament, and Thy-1 expression. However, these exosome recovery functions for the neuro-regeneration markers Neuroflament and Thy-1 did not work in UBA2 knockdown cells. This implied that the exosomes had lost their recovery capability with respect to target proteins in hypoxia-damaged R28 cells. Taken together, these results suggest that UBA2 is a downstream mediator of the recovery pathway induced by exosomes (Fig. 7).

## Discussion

The optic nerve (ON) comprises axons of retinal ganglion cells (RGCs), and this disorder is characterized by RGCs death [26]. ON injury is one of the leading causes of blindness due to RGC degeneration [27]. Various somatic tissue-derived MSCs have proved significant neuroprotective and axogenic effects on RGCs [27, 28]. There have been many reports using R28 cells in terms of RGC injury model to investigate the rescue function of MSCs from ON damage. However there is a limitation of the RGCs impairment model in replacement of RGCs with R28 retinal precursor cells. But R28 cells are immortalized retinal precursor cells that could differentiate into both neuronal and glial cell properties [29]. In a murine oxygen-induced retinopathy model, intravitreal injection of MSC-derived EVs reduced the severity of retinal ischemia [18]. Along with in vivo study, in vitro experiment was performed so that pretreatment with EVs could defend R28 cells against oxygen deficiency situations [30]. So far, R28 cells provide an important experimental system for the various studies of retinal ganglion cells, such as retinal cell differentiation, neuroprotection, and neuronal function [23, 31].

Exosome treatment offers significant potential advantages over cell therapy. Unlike cells, exosomes do not replicate, change phenotype, or actively migrate from the application site, and can hence be manipulated with more accuracy [32]. Moreover, they can be more precisely dosed, because they are non-dividing. Exosomes are gradually being recognized as potential biomarkers for neurodegenerative diseases. For example, spinal cord injury can lead to differential regulation of exosomal miRNAs that control calcium signaling, synaptic function, axon guidance, and axon degeneration [33, 34]. Exosome biology in the visual system is not well-characterized; recent studies used exosomes mostly to detect and monitor ON trauma and disease [32]. Exosomes derived from photoreceptors are highly expressed in patients after rhegmatogenous retinal detachment [35], and exosomes containing specific subsets of miRNAs can serve as biomarkers for glaucoma detection and analysis [36].

Furthermore, it has been reported that bone marrow MSC (BMSC)-derived exosomes exert neuroprotective and axogenic effects on RGCs and that the therapeutic effects of these exosomes diminish after knockdown of Argonaute-2, a key miRNA effector molecule [37]. This implies that the mechanism may be related to miRNA in exosomes. It was demonstrated intravitreal injections of exosomes from MSCs prevent axonal loss and degeneration after mechanical injury and involve in the regeneration of injured retinal ganglion cells [37]. Treatment of primary adult rat cortical neurons with BMSC-derived exosomes promoted neurite outgrowth [38]. The promising neurite outgrowth seen when retinal cultures were treated with BMSC-derived exosomes was corroborated by their efficacy to promote regeneration of GAP-43 axons after optic nerve crush [37]. In this study, they successfully knockdown Ago2 and demonstrated that BMSC exosomes had a considerably muted effect in promoting RGC neuroprotection, axon regeneration/survival, and RGC functional preservation [37].

Intravenous transplantation of MSC-derived exosomes improves neurogenesis, neurite remodeling, and angiogenesis after ischemic brain injury [39, 40]. Therapy based on the delivery of MSC-derived exosomes considerably increased the number of neuroblasts in ischemic regions of the central nervous system [39]. Mesenchymal stromal cell exosomes contain miRNAs, messenger RNAs, and proteins, which can be transferred to recipient cells and thereby modify their characteristics [41]. As miRNAs have an essential role in gene regulation, the miRNAs encapsulated into MSC exosomes have a primary effect on ischemic injury. In addition, it is known that the injection of exosomes extracted from placenta-derived MSCs in hypoxic conditions promotes angiogenesis [13]. MSC exosomes are augmented in some nodes associated with NF-κB signaling, which has been reported to be a significant mediator of angiogenesis [42].

However, other mechanisms seem to be at work in exosomes. Recent studies have demonstrated that MSC-derived exosomes can reduce neuro-inflammation, promote neurogenesis, and improve functional rehabilitation in animal models [43]. A study showed that delivery of exosomes derived from MSCs into a patient with steroid-refractory graft-versus-host disease suppressed pro-inflammatory cytokine secretion and reduced the symptoms associated with the disease [44]. The viruses were thought to exit cells through lysis. In recent times, it has become apparent that they use autophagy pathways for viral release [45]. The cell-to-cell spread of cytoplasmic constituents is thought to require cell lysis. Components of the autophagy pathway have been shown to play a role in the secretion of cytoplasmic signaling proteins [45]. Changes in the autophagy level affect exosomal release. Upon stimulating autophagy, multivesicular bodies fuse more with autophagic vacuoles, resulting in inhibition of exosomal release [46, 47]. The autophagy may control exosome composition and progression in age-related neurodegenerative synucleinopathies [47].

Based on our findings, exosomes seem to exert a unique recovery effect on hypoxia-damaged R28 cells via the UBA2-activated Wnt signaling pathway. To investigate the exosome mechanism during the recovery process in CoCl_2_-damaged R28 cells, we gathered clues on the functions of exosomes, using proteomics, and tried to prove the pathway through functional studies. The identified protein markers validated the accuracy of the proteomic analysis [48]. This analysis confirmed that exosomes derived from hPMSCs had a number of characterized proteins involved in protein-containing complexes and RNA binding for molecular functions. Through proteomic analysis, factors related to ubiquitin and Wnt signaling in R28 cells exposed to hypoxic conditions were derived as candidate targets, and their expression was confirmed through Western blot experiments. Additional proteomic measurements can help characterize the overall proteome, and relative protein quantification can aid in identifying the candidate marker proteins.

The ubiquitin-proteasome system (UPS) consists of ubiquitin-activating enzyme E1, ubiquitin-binding enzyme E2, and ubiquitin protein ligase E3 [49]. The Skp1-cullin 1-F-box (SCF) E3 ligase complexes, the largest family of E3 ligases, comprise cullin, Skp1, and F-box proteins. The SCF E3 ubiquitin ligases play an important role in regulating critical cellular processes that promote the degradation of many cellular proteins, including signal transducers, cell cycle regulators, and transcription factors [50]. Changes in ubiquitin-related factors upon CoCl_2_ exposure confirmed that the expression of UBA2, UBE2I, UBE2E3, and ubiquitin decreased in cells treated with CoCl_2_ and that the expression recovered after exosome treatment.

UBA2 is known to promote cell proliferation. Inhibition of UBA2 expression reduces the proliferation of colorectal cancer and gastric cancer cells regulating cyclinB1, B cell lymphoma-2, and E3 ubiquitin-protein ligase MDM2 [51, 52]. Moreover, UBA2 has functions such as molecular adhesion, movement, and migration [53]. Factors related to Wnt signaling, one of the processes in proteasome degradation, were also present in the DEP list obtained in this proteomics study. Therefore, the expression of UBA2 in R28 cells verified the role of the exosomes. The ubiquitination process was inhibited due to CoCl_2_ injury, and Wnt signaling was deactivated by abnormal ubiquitination. However, PMSC-derived exosomes restored the UBA2 function and activated the Wnt signaling pathway. Cheng et al. reported that UBA2 was needed for cell migration and invasion through Wnt/β-catenin signaling in tumor cell growth. This study suggested UBA2 could regulate the nuclear localization of β-catenin. Silencing of UBA2 caused inhibition of Wnt/β-catenin downstream molecules [53]. Our study also demonstrated that UBA2 was a key mediator protein in the exosome-induced recovery process of regeneration marker expression altered by exposure to hypoxia conditions.

These results were valuable in identifying DEPs and new markers in the hPMSC exosome proteome. However, the relatively small sample size used in this study limits the validity of the outcomes. Additional samples are required for high-throughput analysis to obtain more accurate proteomic data. Full coverage of peptides could not be achieved for complex biologic samples such as exosomes, since the dynamic range was only suitable to detect the most abundant ionized peptides in MS. Other data-independent acquisition methods may help expand the overall detection ability for proteomes and validate the candidate marker proteins identified by relative protein quantification [48]. The establishment of a proteome map and quantitative analysis platform may provide biologists the tools to investigate unknown biological functions [48].

The ubiquitination system is involved in diverse cellular pathways that entail the post-translational modification of proteins. Protein ubiquitination can be reversed by de-ubiquitinating enzymes (DUBs). DUBs are key players in various cellular processes, and several of them are linked to malignancies and neurological diseases [54]. Although the understanding on functions of DUBs at the structural and cellular levels is limited, the possible regulation of DUB activity in various signaling pathways should be considered as a mechanism of biological therapeutics for neurological diseases.

We could demonstrate the neuroprotective effect afforded by hPMSC-derived exosomes. This study was performed only in vitro to evaluate the potential therapeutic effect of exosomes on hypoxia-damaged retinal precursor cells. The neuroprotective effect of exosomes is to be investigated in an ON injury animal model to validate the rescue function in RGCs. Exosomes offer a cell-free alternative to hPMSC therapy, and they can be easily separated, purified, and stored. Exosome treatments lack the risks or difficulties (immune rejection and unwanted proliferation/differentiation) associated with transplanting live cells into vitreous bodies or veins. The ideal timeframe for such treatments is currently unknown; the efficacy of a single or weekly/monthly injections of exosomes should be studied. This study revealed the significant, albeit limited, ON regeneration effect of exosomes extracted from placenta-derived MSCs. Other sources of exosomes can be studied to compare the nerve regeneration effects on hypoxic injury.

## Conclusions

This is the first report on the therapeutic benefit that hPMSC-derived exosomes offer to protect retinal precursor cells after hypoxic injury. We discovered that UBA2 played a key role in activating the Wnt/β-catenin signaling pathway during the recovery process of damaged R28 cells, induced by hPMSC-derived exosomes.

## Data Availability

All data and materials are available upon request.

## Abbreviations

BMSC: Bone marrow-derived stem cells
hPMSCs: Human placenta-derived mesenchymal stem cells
ON: Optic nerve
RGC: Retinal ganglion cells
